# Effects of Dietary Fiber, Crude Protein Level, and Gestation Stage on the Nitrogen Utilization of Multiparous Gestating Sows

**DOI:** 10.3390/ani12121543

**Published:** 2022-06-14

**Authors:** Min Yang, Lun Hua, Zhengyu Mao, Yan Lin, Shengyu Xu, Jian Li, Xuemei Jiang, De Wu, Yong Zhuo, Jiankui Huang

**Affiliations:** 1 Animal Nutrition Institute, Sichuan Agricultural University, Chengdu 611130, China; yang040101@yeah.net (M.Y.); hualun@sicau.edu.cn (L.H.); maozhengyu1995@163.com (Z.M.); linyan936@163.com (Y.L.); shengyu_x@hotmail.com (S.X.); lijian522@hotmail.com (J.L.); 71310@sicau.edu.cn (X.J.); wude@sicau.edu.cn (D.W.); 2 Pet Nutrition and Health Research Center, Chengdu Agricultural College, Chengdu 611130, China; 3 Guangxi Shangda Technology, Co., Ltd., Guangxi Research Center for Nutrition and Engineering Technology of Breeding Swine, Nanning 530105, China

**Keywords:** crude protein, dietary fiber, gestation stage, nitrogen utilization, sows

## Abstract

**Simple Summary:**

Two major concerns for sustainable swine production are the shortage of protein feed resources and the excretion of nitrogen and phosphorus into the environment. Those concerns can be partly alleviated by increasing nitrogen utilization by improving nutrition for pigs. This study observed that a low crude protein and dietary fiber supplemented diet can improve nitrogen utilization and reduce urinary nitrogen excretion. Additionally, late gestating sows have greater nitrogen utilization than those of early gestation, characterized by reduced nitrogen in urine and lower amino acids in serum. These findings offer nutritional solutions for improving nitrogen utilization.

**Abstract:**

To investigate the effects of dietary fiber (DF), crude protein (CP) level, and gestation stage on nitrogen utilization, 28 Landrace-Yorkshire cross gestating sows at parity two were randomly divided into four dietary treatments with seven duplicates of one pig with a repeated-measures design. The diets comprised one with normal crude protein (CP) of 13.3%, one with a low CP diet of 10.1%, and two diets, one with dietary fiber (DF) supplementation of inulin and cellulose at the ratio of 1:1 and one without DF. The total litter size, litter size alive, and newborn birthweight of piglets did not differ between treatment groups. Sows that received high DF levels had greater nitrogen output in feces, lower urinary nitrogen, and increased nitrogen retention. Sows that received a low CP diet had reduced nitrogen excretion in feces and urine, lower nitrogen retention, and an unchanged nitrogen retention ratio. Sows at the late stage of gestation on days 95 to 98 had lower nitrogen excretion in urine and greater nitrogen retention than in the early stage of gestation on days 35 to 38, associated with a significant decrease in serum amino acids in late gestation. Maternal protein deposition was increased by high DF, decreased by low CP, and lower in late gestation compared with early gestation. Collectively, DF improved nitrogen utilization by decreasing urine nitrogen output, and nitrogen utilization increased as gestation advanced.

## 1. Introduction

Improving the utilization of dietary protein can reduce concerns about the shortage of protein feed resources for pig production, and reducing nitrogen excretion both in feces and urine will alleviate concerns about its environmental impact [1]. Nitrogen excretion from gestating sows accounts for up to 20% of the pollution due to the pig industry [1]. It is a common practice to limit nitrogen excretion by reducing crude protein (CP) content in feed for growing pigs and poultry [2], but it remains uncertain whether the low-protein diet could be applied to pregnant sows without negative effects on their reproductive performance. Dietary fiber (DF) is mainly included in the diets for gestating sows to improve the gut fullness, reduce the incidence of constipation and promote gastrointestinal health with the colonization of beneficial commensal microbiota and their physiologically active by-products such as short-chain fatty acids (SCFAs) following fermentation [3]. The level of DF is another regulator of nitrogen balance, which increases the fecal nitrogen excretion while reducing the nitrogen excretion in urine [4,5], but the effect of dietary fiber inclusion at different levels of dietary crude protein on the nitrogen utilization and reproductive performance remains uncertain. Inulin is a water-soluble fiber (SF) belonging to a group of non-digestible carbohydrates called fructans, and it is easily fermented by microbiota in the gut. In contrast, cellulose is a water-insoluble fiber (ISF) poorly fermented by gut microbiota. The combined supplementation of inulin and cellulose to increase both dietary SF and ISF contents has been observed to exert positive effects on ovarian follicular development in gilts [6,7] and litter size [8] in gestating sows. Pregnant sows have greater nitrogen retention than non-pregnant sows, and this is most pronounced in late gestation [9], so there may be a gestational stage-dependent change in nitrogen retention for pregnant sows. However, it remains uncertain whether dietary fiber inclusion in a low-protein diet could exert a gestational stage-dependent effect on the nitrogen balance and reproductive performance. This study hypothesized that a high DF diet could improve the sows’ nitrogen utilization, which might alleviate the negative effects of the low CP diet on their reproductive performance. The objective was to investigate the effects of dietary fiber, protein level, and gestation stage on the nitrogen utilization for multiparous gestating sows with a repeated-measures analysis.

## 2. Materials and Methods

### 2.1. Ethical Considerations

The present trial was conducted at the research center of Sichuan Agricultural University. All the experimental procedures were performed in accordance with animal care and use committee guidelines of the university (Ethics Approval Code: 20174007) and followed national laws and National Research Council (NRC) guidelines for the care and use of laboratory animals.

### 2.2. Animals, Diets, and Design

A total of 32 Landrace × Yorkshire (LY) at parity two, with a similar mean bodyweight of 164.2 kg and farrowing age, were obtained from the research center of Sichuan Agricultural University. They were artificially inseminated twice with fresh pooled semen from three Duroc boars at 12 h and 24 h after their occurrence of standing heat post weaning. Twenty-eight of them became pregnant, and they were randomly allocated to four dietary treatment groups according to a 2 × 2 factorial design with seven sows per group. Treatment effects included two dietary CP levels and two DF levels. The normal protein (NP) diet was formulated with corn, wheat bran, soybean meal, and soybean protein isolate as the main ingredients to provide 13.3% CP and 0.68% total lysine. The low-protein (LP) diet was formulated by reducing the soybean meal and soybean protein isolate while supplementing crystalline amino acids (AA) to provide an equal level of essential amino acids to LP. Inulin and cellulose (Guangxi Shangda Tech Co., Ltd., Nanning, China) were used as DF, adding to both NP and LP rations at 18 g/kg and 18.9 g/kg to increase the SF and ISF content, respectively, in Table 1, with the supplementation of insulin and cellulose increasing the total DF level to 15.8% and the ISF and SF ratio at 6.5:1, as recommended by a recent publication [10]. These four diets contained similar levels of digestive energy at 3.20 Mcal/kg, total lysine, mineral, and vitamins, according to the NRC (2012) [11], and the daily feed intake was 2.4 kg per day throughout gestation. All sows received a similar level of digestive energy, total lysine, mineral, and vitamins except for intakes of CP and DF. The pregnant sows were individually housed in a 0.65 m × 2.20 m gestation stall from day one to 110 of gestation. On day 111 of gestation, sows were transferred to an individual farrowing pen, where they were fed twice daily with equal amounts of feed at 08:00 a.m. and 16:00 p.m. and had free access to water. The average ambient temperature was 20 to 24 °C.

### 2.3. Measurements of Growth and Reproductive Outcomes

Backfat (BF) thickness at 65 mm on the left side of the dorsal mid-line of the last rib (P2) was measured on days one, 30, 90, and 110 of gestation using a digital BF indicator (Renco Lean-Meater, Renco Corporation, Minneapolis, MN, USA). Body weight (BW) was assessed on the same days.

Average daily gain (ADG) and BF gain were calculated by weight gain and BF gain divided by the time in days of the experiment during each experimental period. Farrowing was attended, and the total numbers of piglets born, piglets born alive, and stillborn or mummified were recorded. Piglet birth weight was recorded immediately after birth, prior to suckling. Intrauterine growth retarded (IUGR) piglets were defined as birth weight less than two standard deviations of average birth weight per litter.

### 2.4. Nitrogen Balance Trial

Gestating sows were moved to stainless-steel metabolism crates to measure their nitrogen balance, as previously described [4,12]. There were two nitrogen balance trials conducted on days 30 to 38 and 90 to 98 of pregnancy. Each nitrogen balance trial was performed over a nine-day period, including a five-day adaptation and a four-day sample collection period. The metabolism crates were equipped with water supply and feeders. Gestating sows were provided with half of their daily feed allowance at 08:00 a.m. and 16:00 p.m. and had free access to water. To protect against the direct contamination of feces by the urine, a metal gauze strip was placed between the metabolism cage and the urine-collecting bottle, which allowed urine to pass through while retaining the feces. Sows were excluded from the nitrogen balance trail if urine was contaminated with feces. A 24 h defecation schedule was maintained to collect urine and feces. Chromic oxide was added to feed at the ratio of 5/100 at 08:00 p.m. on the fifth day of each trial to indicate the start of feces collection and to label feces, and on the 10th day to indicate the end of feces collection and to label feces. Feces collected every 24 h were pooled, weighed, and stored on ice. Microbial fermentation of the collected feces was prevented by the immediate addition of several methylbenzene drops, and 1 mL 10% HCl was added to each 10 g of wet feces to prevent nitrogen loss. The urine was collected and measured every 24 h, from 08:00 a.m. on day five to 08:00 p.m. on day 10 during each trial. Sulfuric acid was added to urine to maintain a pH below 3.0. Collected feces and urine samples were pooled at the end of each nitrogen balance trial. Representative urine sub-samples (5%) were collected and stored at 4 °C until analysis.

### 2.5. Chemical Analysis

All diet samples from three batches were pooled, homogenized, and stored at 4 °C until analysis. Amino acid contents in diets were analyzed on an automatic AA analyzer (LA8080; Hitachi Co., Tokyo, Japan). Dry matter contents in feed and feces samples were measured using forced-air oven drying methods (AOAC; method 930.15, 1997). A 150 g fecal sub-sample of seven pigs per group collected at each nitrogen balance trial was freeze-dried and homogenized. These freeze-dried fecal samples, diet samples, and liquid urine sub-samples were used for N content analysis by the Kjeldahl method. Nitrogen balance concentrations were calculated as follows:

Retained nitrogen (g/d) = nitrogen intake (g/d) − nitrogen feces (g/d) − nitrogen urine (g/d)

Absorbed nitrogen (g/d) = nitrogen intake (g/d) − nitrogen feces (g/d)

Nitrogen digestibility (%) = Absorbed nitrogen (g/d) ÷ nitrogen intake (g/d) × 100

Nitrogen utilization (%) = Retained nitrogen (g/d) ÷ absorbed nitrogen (g/d) × 100

Net nitrogen utilization (%) = Retained nitrogen (g/d) ÷ nitrogen intake (g/d) × 100

### 2.6. Serum Amino Acids Level in Sows

Serum samples from fasted sows were collected on days 30 and 90 of pregnancy. The frozen serum samples were thawed on ice. A total of 1 mL of the serum sample was then mixed thoroughly with 1 mL of hexyl hydride and 0.5 mL of 15% (*w*/*v*) sulphosalicylic acid solution and centrifuged at 12,000× *g* at 4 °C for 30 min. The supernatant was collected and analyzed for amino acids by ion-exchange chromatography using an L8800 high-speed AA analyzer (Hitachi, Tokyo, Japan). Each essential and non-essential amino acid was summarized to provide a total of essential amino acids (TEAA) or total non-essential amino acids (TNEAA).

### 2.7. Maternal and Pregnancy-Associated Protein Deposition

Whole-body protein deposition (Pd) was calculated by the nitrogen retention obtained by the nitrogen balance trial. The pregnancy-associated Pd was calculated by the following equations raised by NRC (2012) [11]:

Protein content of empty uterus (g)= exp(6.6361−2.4132×exp(−0.0101×t)),

 Protein content of mammary gland tissue = exp(8.4827−7.1786×exp(−0.0153×(t−29.18))),

Protein content of fetus = exp(8.729−12.5435×exp(−0.0145×t)+0.0867×ls),

$$\begin{array}{cc} \mathrm{Protein}\;\mathrm{content} & \mathrm{of}\;\mathrm{placenta}\;\mathrm{and}\;\mathrm{chorioallantoic}\;\mathrm{fluid}\\ & =\left(38.54\times {\left(t÷54.969\right)}^{7.5036}\right)÷(1+{\left(t÷54.969\right)}^{7.5036}) \end{array}$$

where *t* denotes pregnancy time, and *ls* denotes litter size.

The maternal Pd was calculated as the difference between whole-body Pd and pregnancy-associated Pd for each nitrogen balance period.

### 2.8. Statistical Analysis

In this study, the sow was used as the experimental unit, and Shapiro–Wilk and Levene tests assessed whether the data being analyzed were normally distributed. Growth and reproductive data were analyzed by a mixed procedure approach using SAS 9.4 (SAS Institute Inc., Cary, NC, USA), and the fixed dietary effects were CP level, DF level, and interactions between CP and DF levels. The data for nitrogen balance, Pd, and serum amino acids at different gestational stages were analyzed using the MIXED procedure in SAS 9.4:

*Y*_ijkl_ = *µ* + *α*_i_ + *β*_j_ + *γ_k_* + (*αβγ*)_ijk_ + *t*_Ɩ_ + *Ɛ*_ijkl_

where *Y*_ijkl_ is the response variable, *µ* is the overall mean; *α_i_*, *β_j_*, and *γ_k_* are fixed effects of CP levels (NP or LP), DF levels with or without DF, and day 30 or 90 of gestation. (*αβγ*)_ijk_ is the interaction among fixed effects, pen is the random effect, *t*_Ɩ_ is the random effect, and *ε*_ijkl_ is the residual error. Results were expressed as the mean ± SEM. Differences were considered significant at *p* < 0.05, whereas 0.05 ≤ *p* < 0.10 values indicated a tendency.

## 3. Results

### 3.1. Removal of Sows

One sow from each group was removed due to conception failure, leaving seven in each dietary treatment group. During the nitrogen balance trial, one sow’s urine was contaminated with feces, and it was excluded from data analysis relating to nitrogen balance.

### 3.2. Gilt Growth Traits during Gestation

As shown in Table 2, the ADG on days 91–110 of gestation was affected by the interaction of CP level and DF level (*p* = 0.022). DF level did not affect the ADG of gestating sows fed the NP diet but improved the ADG when sows were fed the LP diet (910.3 g/d vs. 972.3 g/d, *p* = 0.05). The BW, BF thickness, and BF gain at different time periods were not affected by CP level, DF level, or the interaction of CP level and DF level (*p* > 0.05).

### 3.3. Nitrogen Balance

The nitrogen balance of gestating sows as affected by different dietary treatments at different gestational stages is shown in Table 3. Sows receiving high DF levels had greater nitrogen output in feces of 6.7 g/d compared with 6.3 g/d (*p* = 0.094), lower urinary nitrogen at 13.1 g/d compared with 15.9 g/d (*p* = 0.002), increased nitrogen retention of 22.6 g/d (63.1%) compared with 20.4 g/d at 56.2% (*p* = 0.026), lower nitrogen digestibility at 83.9% compared with 85.2 (*p* = 0.002) and greater net nitrogen utilization at 53.1% compared with 47.8% (*p* = 0.027).

Sows receiving low dietary CP levels resulted in lower fecal nitrogen at 6.1 g/d compared with 6.9 g/d (*p* = 0.003), decreased urinary nitrogen at 12.3 g/d compared with 16.7 g/d (*p* < 0.001), lower nitrogen retention at 17.9 g/d compared with 25.2 g/d (*p* = 0.003) and unchanged nitrogen retention ratio and net nitrogen utilization (*p* > 0.05).

The stage of gestation affected the urinary nitrogen, nitrogen retention, nitrogen digestibility, nitrogen retention ratio, and nitrogen net utilization ratio (*p* < 0.05). Total nitrogen output was decreased by high DF and low CP diets and was lower in late gestation on days 95 to 98 compared with early gestation on days 35 to 38. The percentage of nitrogen from urine accounted for approximately 70% of total nitrogen excretion. Urine nitrogen output were lower by high DF level at 65% compared to 71% (*p* = 0.001), low CP level at 66% compared with 70% (*p* = 0.022) and large gestation at 65% compared with 71% (*p* < 0.001).

### 3.4. Serum Amino Acids

The serum AA level of gestating sows as affected by different dietary treatments at different gestational stages is shown in Table 4. Sows that received high DF levels had lower Thr levels (0.66 vs. 0.56 mmol/L, *p* = 0.044) but had no effect on the serum levels of other amino acids and the sum of essential or non-essential AA (*p* > 0.05). Sows that received low CP levels had higher serum levels of Lys and Thr and lower levels of Try, Ile, and Val (*p* < 0.05) but had no effect on the serum levels of other AA (*p* > 0.05). The stage of gestation had a significant effect on the serum levels of both essential AA and non-essential AA. The serum levels of Lys, Try, Thr, Ile, Leu, Val, TEAA, Ser, GluNH_2_, Gly, Orn, Pro, and TNEAA were lower in sows at late gestation than in early gestation (*p* < 0.05 or *p* < 0.01). Each serum AA was not affected by the interactions of DF × CP, DF × Stage, CP × Stage, and DF × CP × Stage (*p* > 0.05).

### 3.5. Whole-Body Pd, Maternal Pd, and Pregnancy-Associated Pd

As shown in Table 5, whole-body Pd were affected by DF level (*p* = 0.026), CP level (*p* < 0.001) and stage of gestation (*p* < 0.001). Pregnancy-associated Pd was only affected by stage of gestation (*p* < 0.001). Maternal Pd was increased by high DF at 112.5 g/d compared with 102.0 g/d (*p* = 0.063), decreased by low CP at 84.3 g/d compared with 130.3 g/d (*p* = 0.001) and lower at 97.5 g/d compared with 117.1 g/d (*p* = 0.033) in late gestation on day 95 to 98 compared with early gestation on day 35 to 38.

## 4. Discussion

Nitrogen metabolism plays an important role in regulating the productive performance of gestating sows, as well as affecting the excretion of nitrogen to the environment, so developing nutritional solutions to improve both would be beneficial. It is a common practice to limit the nitrogen output to the environment by reducing dietary CP content and supplementing with crystalline amino acids to meet the essential amino acid requirements [1]. The normal level of crude protein content for a normal or low CP diet has not been clearly defined, but a normal CP diet usually has low supplementation of crystal amino acids, so lysine would be added, for example, if levels fell below 0.1% for a normal diet [1]. In practice, the CP content in gestation diets usually contains 12 to 14% CP, and a low CP diet is characterized by reduced soybean meal addition and greater supplementation of crystal amino acids. In one study, replacing the dietary soybean meal with crystalline amino acids to decrease the dietary CP by 2% reduced feed costs and nitrogen excretion without negative effects on the growth and health of growing pigs [11]. In the present study, feeding the gestating sows low CP diets did not affect the growth or back fat gain during gestation, suggesting that low protein diets can be applied to gestation without affecting the growth and reproductive performance.

The level of DF was often ignored in pig diets because it was considered to negatively affect the digestion of other nutrients [13]. Supplementation of fiber-rich ingredients to sows was shown to decrease the nutrient digestibility and growth performance, although some beneficial effects of DF have been observed [14,15,16]. In other studies, the combined supplementation of inulin and cellulose to increase both dietary SF and ISF contents had positive effects on ovarian follicular development in gilts [6,7] and litter size [8] in gestating sows. The current findings revealed that DF addition to both normal and low CP diets did not negatively affect the ADG of gestating sows, which is consistent with previous research using pectin, inulin, and cellulose as DF [4]. However, the ADG during days 91 to 110 of gestation was not affected by DF supplementation in sows fed the low CP diet but was increased by DF in gestating sows fed the low CP, indicative of the effect of DF and CP levels on the nutrient utilization of late gestating sows. A recent study observed that a low CP diet did not affect the ADG of gestating sows during days one to 90 of gestation but decreased the ADG at late gestation [4]. Those results indicated that the effects of DF and CP levels on the growth of sows might be dependent on the stage of gestation.

The present study found that a low CP diet resulted in lower fecal and urine nitrogen and reduced nitrogen retention, but at a similar ratio of nitrogen retention at 59.2% compared to 60.1% for gestating sows, but the apparent nitrogen digestibility in the LP group of 83.3% was lower than the NP group at 85.8%. This might be due to the greater percentage of fecal nitrogen at 34% in the LP group compared with 30% for the NP group. Although daily nitrogen intake was reduced by 28%, fecal nitrogen excretion was reduced by 12%. The microbial protein accounted for 80% of total fecal nitrogen [4], so the greater percentage of fecal nitrogen excretion implicated that microbiota plays an important role in the way of nitrogen output [5].

Adding inulin and cellulose as DF sources to the sow’s gestational diet increased the fecal excretion of nitrogen by 0.4 g/d, reducing the apparent digestibility of nitrogen by 1.3%, which was consistent with previous research conducted on rats [17], growing pigs [5] and gestating sows [4,15]. The decrease in nitrogen digestibility might have two reasons. Dietary fiber may be able to lower the activities of digestive enzymes such as pancreatic lipase, trypsin, and chymotrypsin that are required for the digestion of nutrients [18,19], or the microbial activity was greater for gestating sows [8], and microbial protein accounted for 60 to 80% of the nitrogen in the feces [4]. The DF is a carbon source driving microbial growth, which stimulates the gut microbiota to use the dietary protein or endogenous nitrogen to synthesize their protein [20].

This study showed that urinary nitrogen excretion accounts for 65 to 70% of total output and found that a high DF level decreased the urinary nitrogen excretion by 2.79 g/d, and this reduction was greater than the increase in fecal nitrogen output. The retained to absorbed nitrogen retention ratio and net nitrogen utilization ratio were, therefore, greater for the high DF level group than the low DF level group. Similar results have been observed for growing pigs [5,21], but the exact mechanism mediating the effects of DF on urine nitrogen excretion remained uncertain. One possible explanation was that the DF induced growth and proliferation of gut microbiota, which promoted the conversion of blood-borne small-molecule nonprotein waste such as urea to microbial protein [22]. The DF-changed gut microbiota has a greater capacity to use the nonprotein nitrogen to synthesize microbial protein in vitro [4], and the microbial protein can be digested to amino acids by the porcine digestive enzymes, which in turn provides the host with a substantial amount of essential amino acids [23]. However, this hypothesis still needs further investigation.

The present study analyzed the time-dependent changes in nitrogen balance using a repeated-measures analysis. One of the most important findings was that sows in late gestation on days 95 to 98 had lower urine nitrogen excretion than early gestation on days 35 to 38, resulting in a greater nitrogen utilization ratio. In support of this, it was observed that most of the serum amino acids were utilized in late gestation. The fetus grows rapidly at this time, and physiological changes would stimulate the placental nutrient transfer from the dam to the fetus to meet the fetal development [24,25].

Nitrogen retention can be divided into two fractions, including protein deposition (Pd) in maternal tissue and pregnancy-associated tissues. In general, a greater maternal Pd was observed in late gestation than in early gestation, which agreed with the recent findings [9]. Those results suggested that pregnancy anabolism might be related to increased protein accretion during late gestation. This study also found that a low CP diet significantly decreased the maternal Pd but adding DF to the gestational diet resulted in a greater maternal Pd at 112.5 g/d compared with 102.0 g/d compared with the low DF diet. Those findings, combined with the serum amino acid levels and the nitrogen balance data, suggest that DF supplementation with a low CP diet significantly affected the protein metabolism at different gestation stages.

## 5. Conclusions

Supplementation of cellulose and inulin as DF increased fecal nitrogen excretion and decreased urine nitrogen excretion, resulting in greater nitrogen utilization. The late gestating sows had greater nitrogen utilization by reducing urine nitrogen output and amino acids in serum. Based on the current findings, a high DF diet can be applied to diets of gestating sows to improve nitrogen utilization, even if they are fed with a low CP diet.

## Figures and Tables

**Table 1:** Diet composition and nutrient content.

Ingredients	Treatments ^1^			
	NPLF	NPHF	LPLF	LPHF
Corn	735.0	700.0	735.0	700.0
Wheat bran	100.0	90.0	100.0	90.0
Soybean meal (CP 44%)	69.8	69.8	40.8	40.8
Soybean protein isolate	32.0	37.0	0.0	5.0
Corn starch	20.0	0.0	71.5	53.8
Fish meal (CP65%)	9.0	9.0	9.0	9.0
Soybean oil	0.0	23.5	3.5	25.0
Calcium carbonate	11.0	11.0	11.0	11.0
CaHPO_4_	12.5	12.5	12.5	12.5
Sodium chloride	4.0	4.0	4.0	4.0
Inulin (>95%)	0.0	18.0	0.0	18.0
Cellulose (>95%)	0.0	18.9	0.0	18.9
L-Lysine HCl (98.5%)	0.8	0.6	4.0	3.7
DL-Methionine (99%)	0.2	0.2	1.1	1.1
L-Threonine (98.5%)	0.1	0.0	1.5	1.2
L-Tryptophan (98.5%)	0.1	0.0	0.6	0.5
Choline chloride (50%)	1.5	1.5	1.5	1.5
Vitamin-mineral premix ^2^	4.0	4.0	4.0	4.0
Analyzed nutrient compositions				
Calculated DE, Mcal/kg	3.20	3.20	3.20	3.20
CP, %	13.32	13.28	10.13	10.22
SF, %	0.62	2.43	0.60	2.41
ISF, %	12.21	13.43	12.11	13.34
DF, %	12.83	15.86	12.71	15.75
Calcium, %	0.88	0.89	0.88	0.87
Total P, %	0.69	0.68	0.67	0.67
Lysine, %	0.67	0.69	0.66	0.66
Met+Cys, %	0.45	0.46	0.45	0.47
Threonine, %	0.53	0.52	0.51	0.53
Tryptophan, %	0.16	0.15	0.17	0.16

^1^ NPLF, normal protein level with low dietary fiber; NPHF, normal protein level with high dietary fiber; LPLF, low protein level with low dietary fiber; LPHF, low protein level with high dietary fiber. ^2^ The premix provided the following vitamin and trace minerals per kilogram of diet: 4000 IU vitamin A; 440 IU vitamin E; 800 IU vitamin D_3_; 0.5 mg vitamin K; 1.0 mg vitamin B_1_; 3.75 mg vitamin B_2_; 1.0 mg vitamin B_6_; 15 μg vitamin B_12_; 12 mg pantothenic acid; 10 mg niacin; 1.3 mg folic acid; 0.2 mg D-biotin; Fe, 80 mg as ferrous sulfate; Zn, 100 mg as zinc sulfate; Cu, 6.6 mg as copper sulfate; Mn, 30 mg as manganese sulfate; I, 0.3 mg as potassium iodide; Se, 0.3 mg as sodium selenite.

**Table 2:** Effects of dietary treatments on the growth traits of gestating sows.

	Normal CP		Low CP		SEM	*p*-Value		
	Low DF	High DF	Low DF	High DF		CP	DF	CP × DF
BW, kg								
Initial (Day 0)	164.1	164.2	163.7	164.7	3.4	0.987	0.872	0.880
Day 30	180.1	182.9	183.2	182.1	3.7	0.768	0.818	0.616
Day 90	210.8	212.6	214.3	213.9	4.2	0.572	0.874	0.789
Day 110	230.1	231.5	232.5	233.3	4.3	0.627	0.800	0.941
ADG, g/d								
Day 0–30	533.3	624.4	649.4	578.9	79.1	0.660	0.898	0.319
Day 31–90	777.2	806.9	844.4	818.6	53.7	0.471	0.971	0.610
Day 91–110	967.0 ^b^	922.6 ^ab^	910.3 ^a^	972.3 ^b^	21.4	0.869	0.685	0.022
Day 0–110	599.8	612.4	626.1	623.3	29.6	0.536	0.871	0.797
BF, mm								
Initial (Day 0)	18.8	18.8	20.3	19.9	1.0	0.209	0.809	0.840
Day 30	19.4	19.4	21.5	19.9	0.9	0.175	0.401	0.409
Day 90	20.6	20.6	22.9	20.7	1.0	0.265	0.303	0.294
Day 110	20.8	20.8	22.3	21.4	1.1	0.366	0.703	0.688
BF gain, mm								
Day 0–30	0.58	0.61	1.16	0.03	0.282	0.994	0.066	0.055
Day 31–90	1.20	1.24	1.40	0.79	0.337	0.709	0.401	0.349
Day 91–110	0.20	0.20	−0.62	0.70	0.390	0.686	0.106	0.108
Day 0–110	1.98	2.05	1.94	1.52	0.458	0.540	0.700	0.603

CP, crude protein; DF, dietary fiber; those with different superscript letters ^a,b^ indicate *p* < 0.05.

**Table 3:** Effects of dietary fiber and crude protein levels on nitrogen balance of gestating sows at different gestational stages.

	DF Level		CP Level		Stage		SEM	*p*-Value						
	Low	High	LP	NP	D30	D90		DF	CP	S	DF × CP	DF × S	CP × S	DF × CP × S
N intake, g/d	36.3	35.7	30.2	41.9	36.2	35.9	1.3	0.301	<0.001	0.460	0.784	0.271	0.635	0.555
N in feces, g/d	6.3	6.7	6.1	6.9	6.4	6.6	0.2	0.094	0.003	0.459	0.743	0.270	0.634	0.556
N in urine, g/d	15.9	13.1	12.3	16.7	16.5	12.6	0.9	0.002	<0.001	<0.001	0.602	0.643	0.286	0.293
N retention, g/d	20.4	22.6	17.9	25.2	19.7	23.4	1.1	0.026	<0.001	<0.001	0.581	0.459	0.404	0.432
N digestibility, %	85.2	83.9	83.3	85.8	84.8	84.3	0.6	0.071	<0.001	0.508	0.559	0.241	0.717	0.455
N retention, %	56.2	63.1	59.2	60.1	54.5	64.8	2.2	0.008	0.719	<0.001	0.932	0.607	0.778	0.405
N net utilization, %	47.8	53.1	49.3	51.6	46.2	54.7	1.9	0.027	0.315	<0.001	0.832	0.528	0.789	0.497
Total N output, g/d	22.2	19.9	18.4	23.6	22.8	19.2	0.9	0.018	<0.001	<0.001	0.570	0.459	0.404	0.432
Urine N output,%	71	65	66	70	71	65	0.02	0.001	0.022	<0.001	1.000	0.961	0.768	0.110
Fecal N output, %	29	35	34	30	29	35	0.02	0.001	0.022	<0.001	1.000	0.961	0.768	0.110

CP, crude protein; DF, dietary fiber; S, stage of gestation; DF × CP, interaction of DF and CP; DF × S, interaction of DF and S; CP × S, interaction of CP and S; DF × CP × S, interaction of DF, CP, and S. Data are given as means and SEM, *n* = 6–7.

**Table 4:** Effects of dietary fiber and crude protein levels on serum levels of amino acids (mmol/L) in gestating sows at different gestational stages.

	DF Level		CP Level		Stage		SEM	*p*-Value						
	Low	High	LP	NP	D30	D90		DF	CP	S	DF × CP	DF × S	CP × S	DF × CP × S
Essential AA														
Lys	0.49	0.48	0.51	0.46	0.63	0.35	0.02	0.779	0.038	0.001	0.568	0.357	0.828	0.672
Met	0.15	0.15	0.16	0.14	0.17	0.14	0.01	0.985	0.092	0.111	0.028	0.125	0.109	0.930
Try	0.07	0.08	0.07	0.08	0.11	0.05	0.01	0.761	0.648	0.001	0.807	0.981	0.605	0.436
Thr	0.66	0.56	0.63	0.59	0.70	0.52	0.02	0.044	0.094	0.022	0.123	0.563	0.525	0.333
Ile	0.29	0.30	0.26	0.33	0.34	0.25	0.02	0.999	0.031	0.004	0.036	0.655	0.253	0.527
Leu	0.97	1.03	0.97	1.02	1.14	0.86	0.03	0.345	0.414	0.001	0.306	0.847	0.476	0.759
Phe	0.34	0.37	0.35	0.36	0.36	0.35	0.02	0.251	0.542	0.637	0.666	0.646	0.858	0.880
Val	0.84	0.87	0.73	0.98	0.97	0.74	0.03	0.643	<0.001	0.005	0.117	0.944	0.062	0.758
His	0.37	0.38	0.36	0.39	0.41	0.34	0.02	0.810	0.377	0.106	0.648	0.057	0.729	0.529
TEAA	4.11	4.22	4.04	4.30	4.78	3.56	0.13	0.737	0.086	0.004	0.131	0.520	0.428	0.757
Non-essential AA														
Asp	0.10	0.09	0.10	0.09	0.09	0.10	0.01	0.815	0.558	0.549	0.054	0.854	0.450	0.102
Ser	0.55	0.56	0.57	0.54	0.66	0.46	0.03	0.892	0.556	0.004	0.026	0.665	0.941	0.792
Glu	0.69	0.79	0.78	0.71	0.68	0.81	0.04	0.221	0.246	0.111	0.951	0.625	0.922	0.109
GluNH_2_	2.37	2.44	2.43	2.38	3.04	1.77	0.12	0.655	0.754	0.001	0.092	0.264	0.933	0.732
Gly	5.42	5.65	5.55	5.53	6.67	4.40	0.22	0.694	0.957	0.001	0.100	0.407	0.822	0.677
Ala	2.25	2.39	2.37	2.27	2.45	2.16	0.08	0.092	0.368	0.155	0.240	0.129	0.403	0.692
Tyr	0.31	0.30	0.30	0.31	0.31	0.30	0.01	0.937	0.689	0.718	0.056	0.773	0.874	0.494
Orn	0.27	0.31	0.28	0.30	0.36	0.22	0.02	0.187	0.570	0.010	0.349	0.236	0.979	0.963
Pro	0.94	1.13	1.01	1.06	1.21	0.86	0.04	0.113	0.490	0.037	0.934	0.506	0.418	0.758
TNEAA	13.47	13.21	13.48	13.20	15.88	10.81	0.41	0.781	0.697	<0.001	0.205	0.416	0.857	0.753

CP, crude protein; DF, dietary fiber; S, stage of gestation; DF × CP, interaction between DF and CP; DF × S, interaction of DF and S; CP × S, interaction of CP and S; DF × CP × S, interaction of DF, CP, and S. Data are given as means and SEM, *n* = 6–7.

**Table 5:** Effects of dietary fiber and crude protein levels on protein deposition (g/d) in gestating sows at different gestational stages.

	DF Level		CP Level		Stage		SEM	*p*-Value						
	Low	High	LP	NP	D30	D90		DF	CP	S	DF × CP	DF × S	CP × S	DF × CP × S
Whole-body Pd	127.5	141.3	111.8	157.5	123.1	146.2	6.9	0.026	<0.001	<0.001	0.581	0.459	0.404	0.432
Pregnancy-associated Pd	28.8	28.7	28.9	28.6	6.4	51.1	0.14	0.630	0.273	<0.001	0.296	0.637	0.276	0.297
Maternal Pd	102.0	112.5	84.3	130.3	117.1	97.5	4.5	0.063	0.001	0.033	0.720	0.950	0.051	0.739

CP, crude protein; DF, dietary fiber; S, stage of gestation; DF × CP, interaction between DF and CP; DF × S, interaction of DF and S; CP × S, interaction of CP and S; DF × CP × S, interaction of DF, CP, and S. Data are given as means and SEM, *n* = 6–7.

## Data Availability

Not applicable.
